# Relationship between finger movement characteristics and brain voxel-based morphometry

**DOI:** 10.1371/journal.pone.0269351

**Published:** 2022-10-07

**Authors:** Junpei Sugioka, Shota Suzumura, Katsumi Kuno, Shiori Kizuka, Hiroaki Sakurai, Yoshikiyo Kanada, Tomohiko Mizuguchi, Izumi Kondo

**Affiliations:** 1 Department of Rehabilitation Medicine, National Center for Geriatrics and Gerontology, Obu, Aichi, Japan; 2 Faculty of Rehabilitation, School of Health Sciences, Fujita Health University, Toyoake, Aichi, Japan; 3 IoT Innovation Department, New Business Producing Division, Maxell, Ltd. Yokohama, Kanagawa, Japan; Jikei University School of Medicine, JAPAN

## Abstract

**Background:**

Aging is the most significant risk factor for dementia. Alzheimer’s disease (AD) accounts for approximately 60–80% of all dementia cases in older adults. This study aimed to examine the relationship between finger movements and brain volume in AD patients using a voxel-based reginal analysis system for Alzheimer’s disease (VSRAD) software.

**Methods:**

Patients diagnosed with AD at the Center for Comprehensive Care and Research on Memory Disorders were included. The diagnostic criteria were based on the National Institute on Aging-Alzheimer’s Association. A finger-tapping device was used for all measurements. Participants performed the tasks in the following order: with their non-dominant hand, dominant hand, both hands simultaneously, and alternate hands. Movements were measured for 15 s each. The relationship between distance and output was measured. Magnetic resonance imaging measurements were performed, and VSRAD was conducted using sagittal section 3D T1-weighted images. The Z-score was used to calculate the severity of medial temporal lobe atrophy. Pearson’s product-moment correlation coefficient analyzed the relationship between the severity of medial temporal lobe atrophy and mean values of the parameters in the finger-tapping movements. The statistical significance level was set at <5%. The calculated p-values were corrected using the Bonferroni method.

**Results:**

Sixty-two patients were included in the study. Comparison between VSRAD and MoCA-J scores corrected for p-values showed a significant negative correlation with the extent of gray matter atrophy (r = -0. 52; p< 0.001). A positive correlation was observed between the severity of medial temporal lobe atrophy and standard deviation (SD) of the distance rate of velocity peak in extending movements in the non-dominant hand (r = 0. 51; p< 0.001).

**Conclusions:**

The SD of distance rate of velocity peak in extending movements extracted from finger taps may be a useful parameter for the early detection of AD and diagnosis of its severity.

## Introduction

Aging is the most significant risk factor for dementia. Since the latter half of the 20th century, amidst global aging, the average life expectancy of Japanese men and women has reached 84.5 years and 90.6 years, respectively [1]. In the future, the number of people with dementia will increase as the population ages. It is difficult to cure dementia using current medical science; however, early diagnosis and intervention can potentially prevent dementia [2]. Alzheimer’s disease (AD) accounts for approximately 60–80% of all cases of dementia in older adults [3]. It was reported that approximately 50% of patients with mild cognitive impairment (MCI), which falls between dementia and being healthy, will progress to AD within 5 years [4]. Therefore, early AD detection is crucial to prevent the onset and progression of dementia.

Against this social background, several studies have been conducted on the early diagnosis of AD, and it has become possible to diagnose dementia early to some extent. Diagnosing dementia includes imaging tests, such as magnetic resonance imaging (MRI) [5], single-photon emission computed tomography [6], fluorodeoxyglucose-positron emission tomography [7], and cerebrospinal fluid biomarkers [8]. Particularly, voxel-based specific regional analysis systems for Alzheimer’s disease (VSRAD) have recently gained attention as tools to diagnose AD early. VSRAD is a software program that automatically calculates the degree of atrophy in the medial temporal and dorsal brainstem from MR images [9,10]. A study using VSRAD has reported that the diagnostic accuracy of AD improved by using VSRAD and the Mini-Mental State Examination (MMSE) for combined diagnosis [11]. Additionally, an observational study of community-dwelling older adults has shown that the degree of medial temporal atrophy (Z-score) is related to the amount of activity [12]. The Montreal Cognitive Assessment (MoCA) is becoming increasingly popular in clinical practice as a superior screening test for detecting mild AD and MCI. Although MoCA is said to be more sensitive and specific than MMSE for detecting patients with mild AD or MCI [13], there are no reports on the association between VSRAD and MoCA.

It has recently been reported that motor impairment can be detected in the early stages of dementia and MCI, and signs of dementia may be observed from motor impairment. Verghese et al. [14] have conducted a quantitative gait evaluation and reported a decrease in walking ability, including walking speed and stride length, in patients with MCI compared to healthy older adults. We have conducted preliminary studies on finger movements in patients with dementia based on the fact that hand movements may detect pathological changes in the brain at an early stage. Accordingly, we detected finger movement features with cognitive decline; finger dexterity declines at AD and MCI stages compared to healthy conditions [15–19]. In a study of cognitive function and hand function at other institutions, it was reported that the number of finger taps decreased and that the tapping interval increased in patients with AD and MCI than in healthy older adults [20]. The number of studies examining hand function in patients with AD or MCI is increasing [21,22]. However, no reports have examined the relationship between finger function and brain imaging in patients with dementia. It is assumed that finger movements are intricately related to various brain parts and that various aspects have not been investigated yet.

Thus, this study aimed to examine the relationship between finger movements and brain volume in patients with AD using VSRAD software and explore the relationship between brain voxel-based morphometry and cognitive function.

## Materials and methods

### Research design and subjects

This exploratory, cross-sectional study was conducted at the National Center for Geriatrics and Gerontology. This study included patients diagnosed with AD at the Center for Comprehensive Care and Research on Memory Disorders. The diagnostic criteria for AD were based on those provided by the National Institute on Aging-Alzheimer’s Association [23]. AD was diagnosed according to this process: (1) medical examination by a dementia specialist, (2) comprehensive geriatric assessment and neuropsychological examination (Frontal Assessment Battery, Raven’s Colored Progressive Matrices, Alzheimer’s Disease Assessment Scale), (3) brain imaging (magnetic resonance imaging, single-photon emission computed tomography), and (4) electrocardiogram and blood tests (a dementia specialist made a comprehensive judgment to diagnose AD based on the results of these tests). Comprehensive geriatric assessment included Mini-Mental State Examination-Japanese, Barthel Index, Dementia Behavior Scale, Geriatric Depression Scale, Vitality Index, Mini Nutritional Assessment, physical measurements, grip strength, gait speed and Timed Up & Go Test. If a definite diagnosis could not be reached, the diagnosis was discussed at a conference of specialists in dementia and subsequently made. The exclusion criteria were impaired consciousness; tremor; parkinsonism; higher brain dysfunction, such as aphasia or apraxia; epilepsy; paralysis; sensory disturbance; finger dexterity impairment; severe cognitive dysfunction that made neuropsychological testing difficult; left-handedness; and difficulty in MRI measurements.

### Ethical considerations

This study was conducted with the approval of the Ethics and Conflict of Interest Committee of the National Center for Geriatrics and Gerontology (approval number 1485–2). The purpose of this study was explained in advance to the subjects, orally and in writing, and only those who provided consent were included in the study.

### Finger-tapping measurement and cognitive function assessment

**Fig 1:**
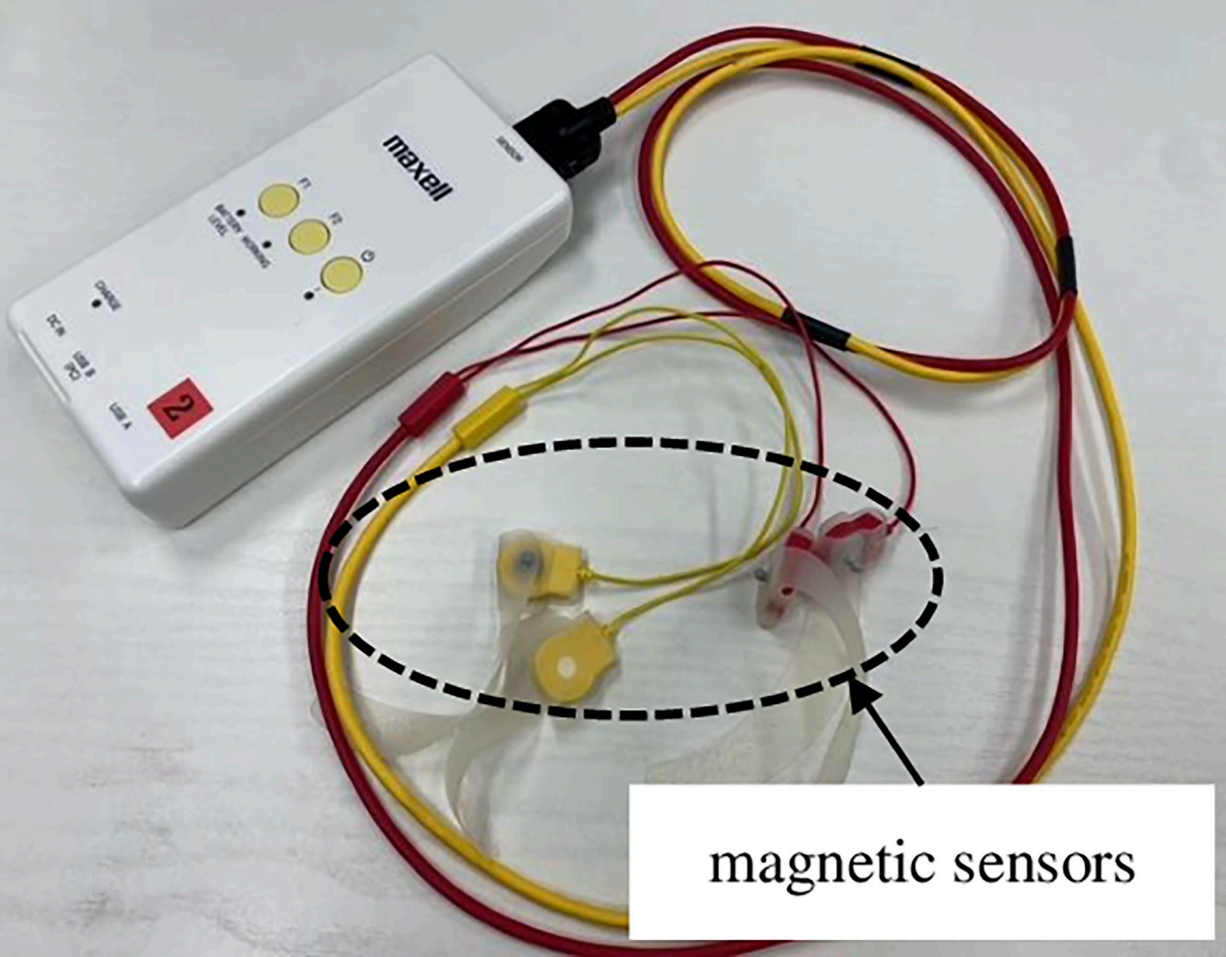
The UB-2 finger-tapping device with magnetic sensors. Yellow cables were used for the left-hand side, and red cables were used for the right-hand side. The magnetic sensor was attached to the dorsal surface of the thumb and index finger and fixed with a rubber belt.

**Fig 2:**
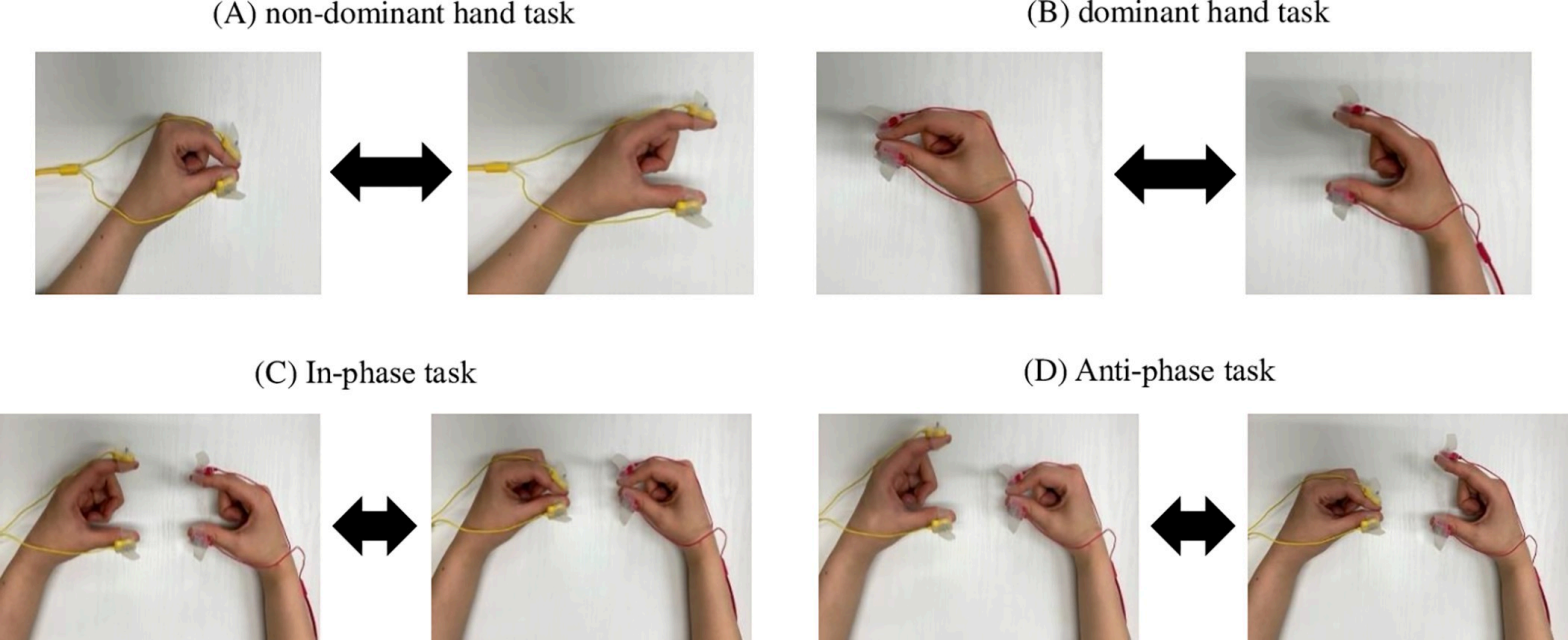
Finger-tapping movements. (A) A tapping action with the left thumb and index finger is performed. (B) A tapping action with the right thumb and index finger is performed. (C) Tapping with the in-phase task is performed. (D) Tapping with the anti-phase task is performed. The distance between the thumb and index finger is kept between 3 and 4 cm, and movement was performed as fast as possible for 15 s.

**Fig 3:**
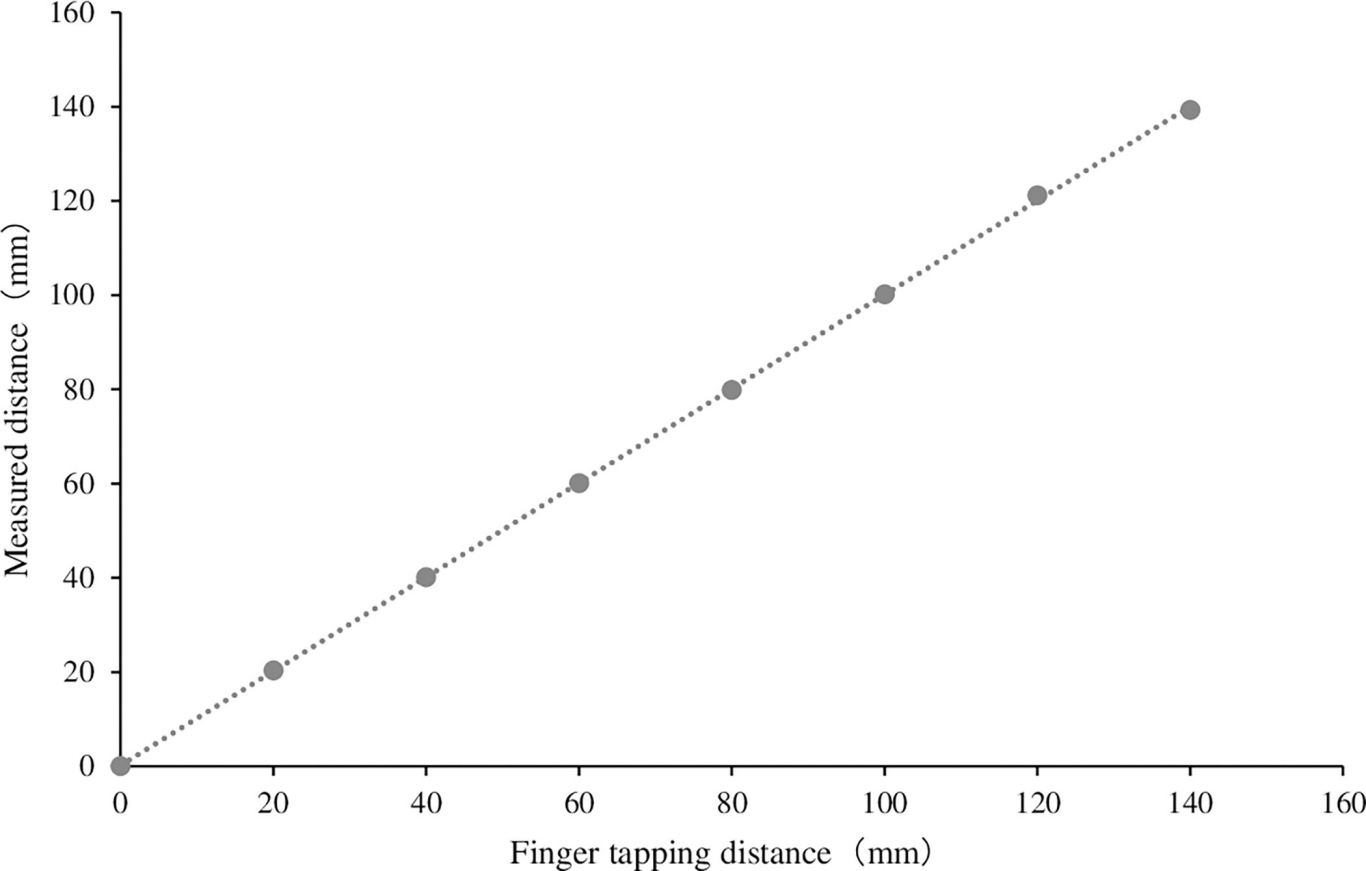
Output characteristics of the measuring equipment. Fig 3 shows the relationship between the finger-tapping distance (mm) and the measured distance (mm) of the device. The slope of the regression line and the R^2^ value were 0.9991 and 0.9999, respectively.

Finger function was measured as finger-tapping movements. Finger-tapping movements were defined as repetitive opening and closing movements by the thumb and index finger. We used a finger-tapping device with magnetic sensors (UB-2, Maxell Holdings, Ltd., Tokyo, Japan) for the measurements (Fig 1). Magnetic sensors were attached to the dorsal surface of the thumb and index finger, and finger-tapping movements were measured. Measurements were performed in the following order: non-dominant hand, dominant hand, both hands simultaneously, and alternate hands. Movements were measured for 15 s (total time for all four movements, 60 s) (Fig 2). The magnetic sensor finger-tapping device can calculate 44 parameters after measuring and recording the finger-tapping motion at 0.1-s intervals (Table 1). The relationship between distance and output was measured as the output characteristics of the device. The slope of the regression line and R^2^ value were 0.9991 and 0.9999, respectively (Fig 3).

**Table 1:** Parameters of finger-tapping movements.

1.	Max distance amplitude (mm)	23.	Max of acceleration amplitude (m/s^2^)
2.	Total traveling distance (m)	24.	Ave of local max acceleration in extending movement (m/s^2^)
3.	Ave of local max distance (mm)	25.	Ave of local min acceleration in extending movement (m/s^2^)
4.	SD of local max distance (mm)	26.	Ave of local max acceleration in flexing movement (m/s^2^)
5.	Slope of approximate line of local max points (mm/s)	27.	Ave of local min acceleration in flexing movement (m/s^2^)
6.	CV of local max distance	28.	Ave of contact duration (s)
7.	SD of local max distance in three adjacent taps (mm)	29.	SD of contact duration (s)
8.	Max of velocity amplitude (m/s)	30.	CV of contact duration
9.	Ave of local max velocity (m/s)	31.	Number of zero crossover points of acceleration
10.	Ave of local min velocity (m/s)	32.	Number of freezing calculated from acceleration
11.	SD of local max velocity (m/s)	33.	Number of taps
12.	SD of local min velocity (m/s)	34.	Ave of tapping interval (s)
13.	Energy balance	35.	Frequency of taps (Hz)
14.	Total energy (m^2^/s^2^)	36.	SD of inter-tapping interval (s)
15.	CV of local max velocity	37.	CV of inter-tapping interval
16.	CV of local min velocity	38.	Inter-tapping interval variability (mm^2^)
17.	Number of freezing calculated from velocity	39.	Skewness of inter-tapping interval distribution
18.	Ave distance rate of velocity peak in extending movement	40.	SD of inter-tapping interval in three adjacent taps (s)
19.	Ave distance rate of velocity peak in flexing movement	41.	Ave phase difference between the left hand and right-hand tapping (degree)
20.	Ratio of distance rates of velocity peak in extending and flexing movements	42.	SD of phase difference between the left hand and right-hand tapping (degree)
21.	SD of distance rate of velocity peak in extending movements	43.	Similarity of hands
22.	SD of distance rate of velocity peak in flexing movement	44.	Time lag of similarity of hands (s)

Forty-four parameters can be calculated from the finger-tapping motion. For numbers 41–44, only the in-phase two-handed task movements were measured.

Abbreviations: Max, maximum; min, minimum; Ave, average; SD, standard deviation; CV, coefficient of variation

Additionally, the Japanese version of the MoCA (MoCA-J) [13], which can quantitatively evaluate the severity of cognitive function in general, was given to all participants.

### Brain MRI measurement and data acquisition

MRI measurements were performed using an Ingenia Ambition 1.5T scanner (Philips Japan, Tokyo). After MRI measurements, a VSRAD analysis (VSRAD advance 2, Eisai Co., Ltd, Tokyo, Japan) was performed using sagittal section 3D T1-weighted images. VSRAD is a software program that measures brain atrophy by volume and automatically calculates the degree of atrophy in the medial temporal region and dorsal brainstem by computing image information obtained by MRI [9,10]. VSRAD can be measured simply by adding the imaging conditions for analysis to the normal MRI imaging and can be performed in <10 minutes with little burden on the human body and no additional charges on insurance. The indices were divided into the gray and white matter. The gray matter indices were calculated as follows: (1) degree of atrophy within the volume of interest (VOI), (2) percentage of total brain atrophy, (3) percentage of atrophy within VOI, (4) atrophy ratio (VOI/total brain atrophy percentage), and (5) maximum value within VOI. The white matter indices were calculated as the percentage of total brain atrophy (Table 2). The region of interest to be used as a reference to support the assessment of brain atrophy in patients with AD was the medial temporal cortex (hippocampus, amygdala, and most of the olfactory cortex). The severity of medial temporal lobe atrophy, considered a region of interest, was calculated using the Z-score. Z score was calculated as follows:

$$Zscore=\frac{control\;group\;average\;voxel\;value-subject\;voxel\;value}{control\;group\;standard\;deviation}$$

**Table 2:** Results calculated by VSRAD.

Item	Parameter	Total value	Right side value	Left side value	Right—Left side
Gray matter ①	Severity of medial temporal lobe atrophy	○	○	○	○
Gray matter ②	Extent of GM atrophy (%)	○	−	−	−
Gray matter ③	Extent of medial temporal lobe atrophy (%)	○	○	○	○
Gray matter ④	Ratio of medial temporal lobe atrophy/GM atrophy (times)	○	−	−	−
Gray matter ⑤	Max in medial temporal lobe atrophy	○	○	○	○
White matter	Extent of WM atrophy (%)	○	−	−	−

○, calculable; -, not calculated.

Gray matter ① Average of medial temporal lobe atrophy above 0.

Gray matter ② Percentage of areas with medial temporal lobe atrophy >2 within the total gray matter.

Gray matter ③ Percentage of regions in medial temporal lobe atrophy of >2.

Gray matter ④ Percentage of cases with a total brain atrophy value of 1.

White matter Percentage of areas with atrophy >2 within the total white matter.

Voxel values are the gray matter and white matter volume density of each voxel expressed in terms of brightness (luminance). One voxel is 2 mm cubic (2 mm x 2 mm x 2 mm). The severity of medial temporal lobe atrophy indicates how much the standard deviation (SD) is separated from the mean value by statistically comparing the subject’s brain image and a normal brain image. As a guide, severity of medial temporal lobe atrophy of 0–1 shows almost no atrophy in the region of interest, severity of medial temporal lobe atrophy 1–2 shows some atrophy in the region of interest, and severity of medial temporal lobe atrophy of 2–3 shows considerable atrophy in the region of interest. If the value is >3, it is judged that the severity of medial temporal lobe atrophy is strong. The percentage of whole brain atrophy in gray matter indicates what percentage of voxels in the whole gray matter are atrophic. As a reference value, atrophy of the whole brain is considered strong when 10% or more is present. The percentage of atrophy within the VOI indicates the extent of medial temporal atrophy; it indicates the percentage of voxels in the medial temporal region that are atrophic. As a reference value, 0–30 indicates a slightly smaller area of atrophy, 30–50 indicates a slightly larger area of atrophy, and ≥50 indicates a larger area of atrophic surface precision. The atrophy ratio (VOI/total brain atrophy percentage) indicates selective atrophy in the medial temporal region. The more selective the atrophy of the area of interest is relative to the atrophy of the whole brain, the greater this value will be. It is a ratio of whole brain atrophy to 1. As a reference, values 0–5 do not indicate selectivity, 5–10 indicate selectivity, and ≥10 indicate strong selectivity. The maximum value within VOI indicates the maximum value of atrophy in the medial temporal lobe. The percentage of whole brain atrophy in the white matter indicates what percentage of voxels in the whole white matter show atrophy. The results of the VSRAD analysis were obtained from MRI data performed in an outpatient memory clinic.

### Statistical analysis

The association between the values calculated by the VSRAD analysis and MOCA-J was analyzed using Spearman’s rank correlation coefficient. After the finger-tapping movements were measured, the measured values of all 44 parameters were calculated from the just tap. Pearson’s product-moment correlation coefficient was used to analyze the relationship between the severity of medial temporal lobe atrophy and the mean values of the parameters in the finger-tapping movements. The calculated p-values were corrected using the Bonferroni method. The statistical significance level was set at <5%. Statistical analysis was performed using SPSS Statistics version 26.0 (IBM Corp., Armonk, NY, USA).

## Results

### Participants’ characteristics

Measurements were performed on 68 individuals. Six left-handed participants were excluded due to the exclusion criteria, and 62 were analyzed. Additionally, five patients could not perform the MoCA-J test due to difficulty understanding the instructions. Thus, 57 patients were included in the MoCA-J analysis (Table 3). Five patients had difficulty performing the MoCA-J analysis. Thus, 57 patients were finally analyzed.

**Table 3:** Patients’ characteristics.

Variables	AD (N = 62)	
		Median (IQR)
Age (years)	76.7 (7.7)	
Sex (%)		
Male	24 (38.7)	
Female	38 (61.3)	
Education (years)	10.7 (2.1)	10 (9–12)
MoCA-J (/30 points) MMSE-J (/30 points) FAB (/18 points) RCPM (/36 points) ADAS (/70 points)	15.7 (5.6) 19.3 (5.4) 8.6 (3.1) 21.4 (8.3) 18.4 (6.8)	17 (12–19) 20 (16–24) 9 (6–11) 24 (16–27) 17.6 (15–21)
Barthel Index (/100 points) Severity of medial temporal atrophy	95.7 (8.4) 1.9 (0.7)	100 (95–100) 1.9 (1.3–2.3)

Characteristics are presented as mean (SD) or n (%).

MOCA-J: Montreal Cognitive Assessment-Japanese.

MMSE-J: Mini-Mental State Examination.

FAB: Frontal Assessment Battery.

RCPM: Raven’s Colored Progressive Matrices.

ADAS: Alzheimer’s Disease Assessment Scale.

### Relationship between VSRAD and MoCA-J

**Fig 4:**
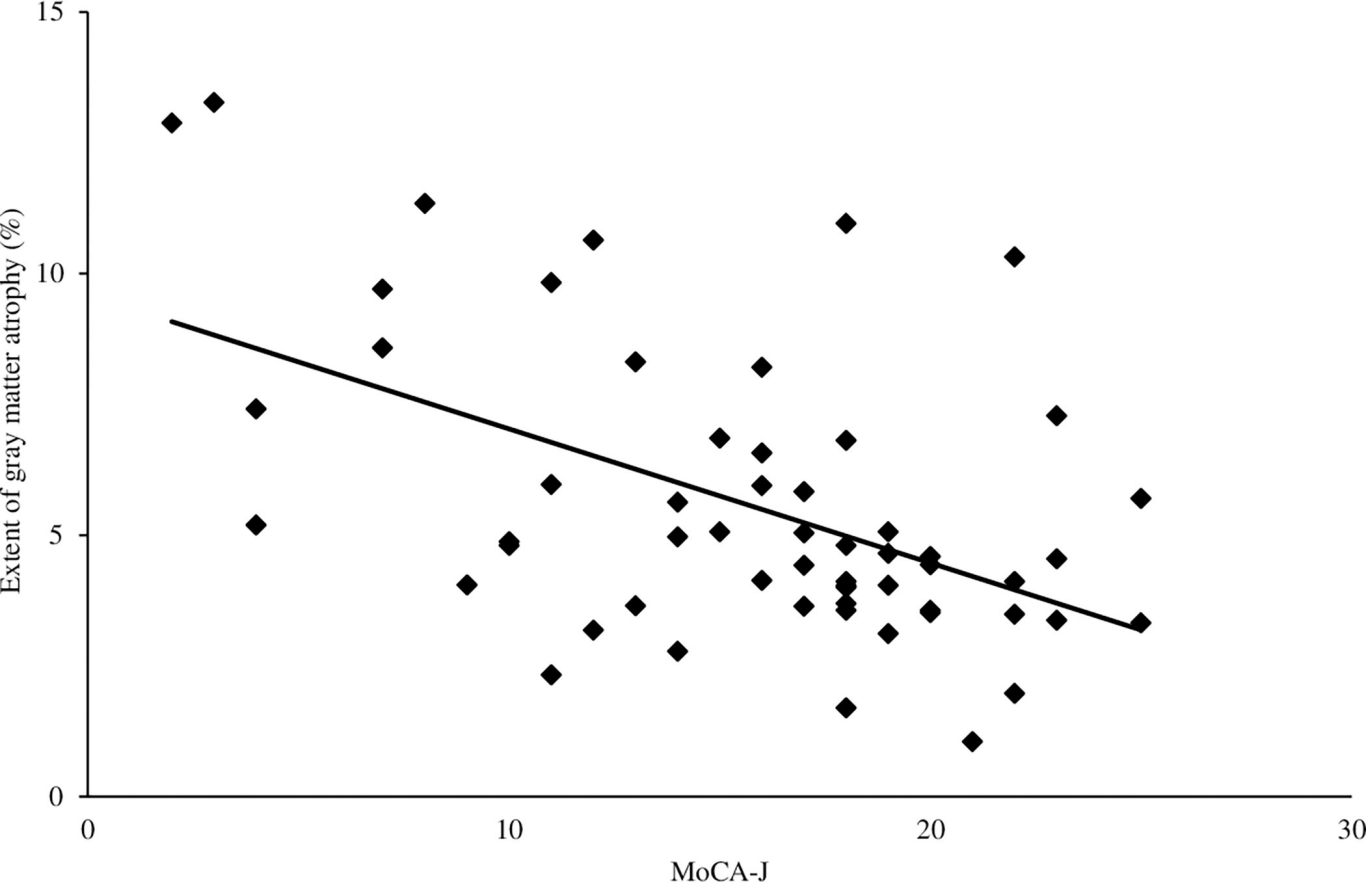
**Relationship between the VSRAD analysis index and MoCA-J.** The vertical axis indicates the VSRAD analysis index, and the horizontal axis indicates the MoCA-J scores. The extent of gray matter atrophy was r = -0.52 and p <0.001.

In relation to the MoCA-J score and VSRAD parameters, only the extent of gray matter atrophy (r = -0.52; p<0.001) showed a significant negative correlation, even after correction for p-values using the Bonferroni method (Fig 4).

### Relationship between severity of medial temporal lobe atrophy and finger-tapping movements

**Fig 5:**
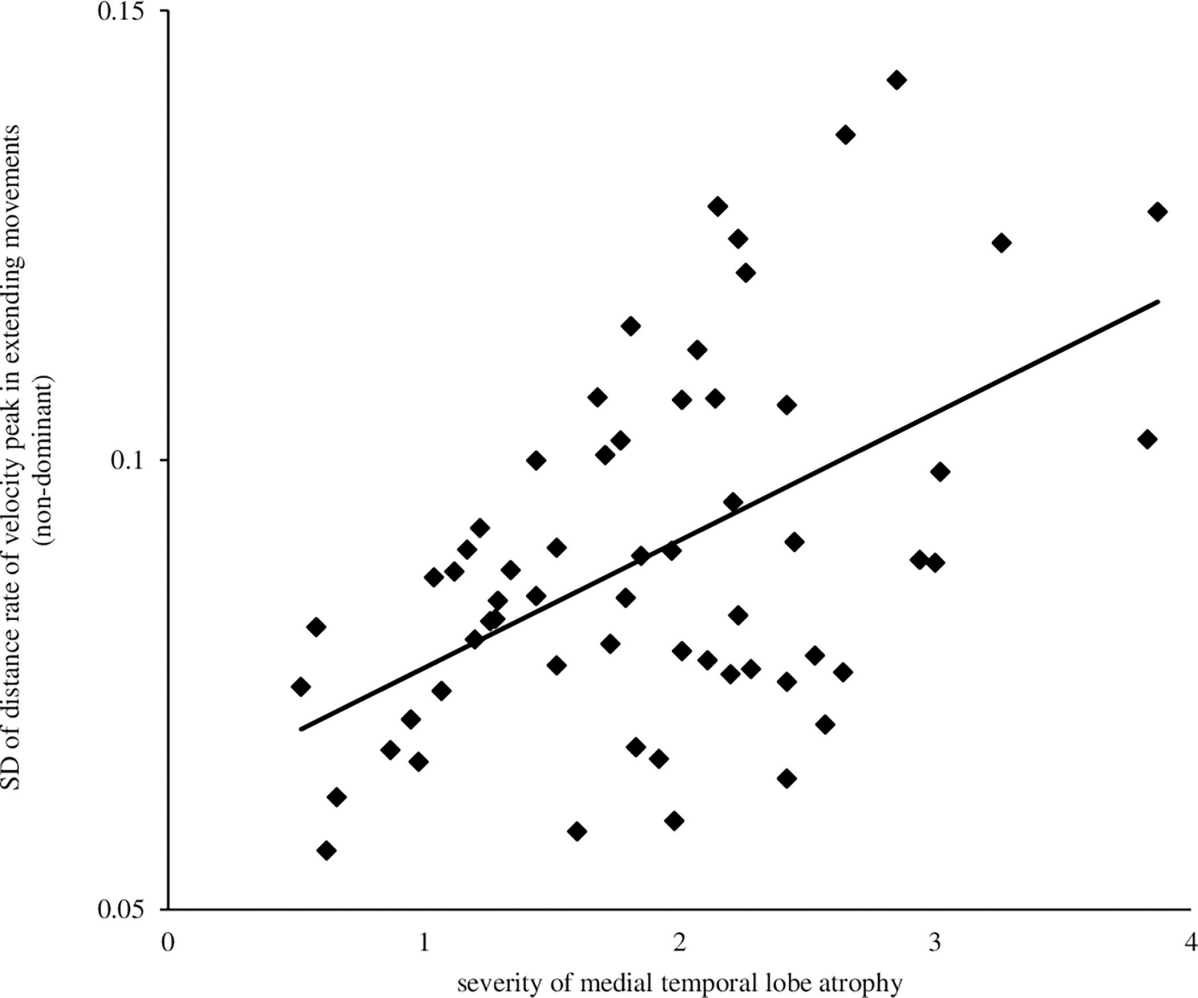
Relationship between finger-tapping parameters and severity of medial temporal lobe atrophy. The vertical and horizontal axes indicate the parameters of the finger-tapping movements and severity of medial temporal lobe atrophy. The SD of distance rate of velocity peak in extending movements of the non-dominant hand was r = 0.51 and p<0.001. The SD of distance rate of velocity peak in extending movements is defined as the SD of the value calculated as the position of the distance at which the velocity is maximal during the finger opening movement as a ratio to the amplitude.

In relation to the severity of medial temporal lobe atrophy and finger tapping parameters, only the SD of distance rate of velocity peak in the extending movements of the non-dominant hand (r = 0.51; p<0.001) remained as a significant positive correlation after correction for p-value using the Bonferroni method (Fig 5). No significant correlations were found between the other finger-tapping parameters and the severity of medial temporal lobe atrophy.

## Discussion

We examined the relationship between finger function, brain function, and cognitive function in patients with AD using VSRAD. The results showed a significant correlation between the SD of distance rate of velocity peak in extending movements and severity of medial temporal lobe atrophy (r = 0.51; p<0.001). The MoCA-J analysis revealed a significant　negative correlation between the extent of gray matter atrophy (r = -0.52; p<0.001).

Although finger movements have been reported to be associated with various brain areas and are still unclear in several cases, to our knowledge, no reports have verified the relationship between finger function and brain imaging analysis in patients with dementia. Although the Brodmann area is well known for its functional localization in the brain, the areas that control finger functions are related to the functions of the primary motor cortex (BA4), premotor cortex, and supplementary motor area (BA6). In addition to these areas, other brain areas, such as the primary somatosensory cortex (BA3, BA1, and BA2) and cerebellum, are involved in a complex manner in finger movement [24–26]. Conversely, the hippocampus, amygdala, and entorhinal cortex participate in motor speed and acceleration during visuomotor tasks and memory and emotion [27,28]. Areas other than the motor cortex, such as the medial temporal region, are further involved in the movement; therefore, brain atrophy due to AD [29] may affect finger movements.

This study found a significant correlation between the SD of distance rate of velocity peak in extending movements and the severity of medial temporal lobe atrophy. This parameter is the variation in the point of maximum velocity while opening the fingers, calculated as the ratio of the point of the maximum velocity to the amplitude. Moreover, it is attributed to changes in the speed of motion. That is, the higher the degree of atrophy of the medial temporal region, the more the movement speed adjustment is affected; therefore, the variability of fingers may increase with atrophy in the VOI. This parameter is similar to the findings from a study mentioned above [18]. The results of this study indicate that this parameter can reflect the aggravation of cognitive function.

Several studies have reported the relationship between the medial temporal cortex, including the hippocampus, and neuropsychological test results in patients with AD. There have been several reports on the relationship between the medial temporal cortex and MMSE, which is known for general cognitive function tests, the Hasegawa Simple Intelligence Scale [30], and the Alzheimer’s Disease Rating Scale [31]; however, no reports have examined the relationship between VSRAD and MoCA-J. This study found the negative correlation between the extent of gray matter atrophy and MoCA-J. In the medial temporal cortex, such as the entorhinal cortex, atrophy has been reported to appear in the early stages of AD and MCI [32–34]. Furthermore, previous studies have reported a correlation between MMSE, a general cognitive function test, and the severity of medial temporal lobe atrophy [30]. In the present study, no significant correlation was found between the severity of medial temporal lobe atrophy and MoCA-J; however, a significant negative correlation was noted with the extent of gray matter atrophy. The overall gray and white matters of the brain are believed to decrease with age [35]. The MoCA-J generally reflects various functions of the brain, and we believe that it could be correlated with the extent of gray matter atrophy. In a previous study, a correlation was obtained between the severity of medial temporal lobe atrophy and the MMSE; however, in our study, no correlation was obtained with the MoCA-J, a similar screening test. We believe that this may be due to the fact that the MoCA-J incorporates more challenging tasks than the MMSE and that the MoCA-J is more specific for MCI than for AD.

This study had some limitations. First, the sample size was small, and the study was conducted in a single center; hence, the influence of selection bias cannot be denied. Therefore, further multicenter studies with more participants are required. Second, this study did not examine patients with MCI at the pre-stage of AD. Several changes to the brain have been reported since MCI onset, such as atrophy of the entorhinal cortex [33,34] and a reduction in gray matter and white mass throughout the brain [36]. Therefore, it is crucial to examine the relationship between the brain and hand function at the MCI stage in the future.

This study examined the relationship between VSRAD, cognitive function, and finger function in patients with AD. The results showed a significant relationship between the SD of distance rate of velocity peak in extending movements, a parameter of the finger-tapping motion, and the severity of medial temporal lobe atrophy. Additionally, we found an association between neuropsychological tests and the overall degree of brain atrophy. The main significance of this study is that it suggests that the SD of distance rate of velocity peak in extending movements extracted from finger taps may be a useful parameter to detect AD early and diagnose its severity. Even if MRI scans cannot be performed, detecting cognitive decline from simple movements such as finger-tapping exercises would be clinically significant. We aim to expand our analysis to include MCI and community-dwelling older adults and examine whether finger-tapping exercise is a useful tool for early detection in the future.

## Supporting information

S1 Table Results of finger tapping (dominant hand). AD, Alzheimer’s disease; max, maximum; min, minimum; Ave, average; SD, standard deviation; CV, coefficient of variation
(DOCX)

S2 Table Results of finger tapping (non-dominant hand). AD, Alzheimer’s disease; max, maximum; min, minimum; Ave, average; SD, standard deviation; CV, coefficient of variation
(DOCX)

S3 Table Results of finger tapping (in-phase). AD, Alzheimer’s disease; max, maximum; min, minimum; Ave, average; SD, standard deviation; CV, coefficient of variation. Upper values represent those for the in-phase left hand. Lower values represent those for the in-phase right hand. Numbers 41–44 are parameters for both-hand tasks only.
(DOCX)

S4 Table Results of finger tapping (anti-phase). AD, Alzheimer’s disease; max, maximum; min, minimum; Ave, average; SD, standard deviation; CV, coefficient of variation. Upper values represent those for the anti-phase left hand. Lower values represent those for the anti-phase right hand. Numbers 41–44 are parameters for both-hand tasks only.
(DOCX)

S5 Table Results of VSRAD analysis. Displays the mean, standard deviation, and interquartile range for each indicator.
(DOCX)

S1 Data Data of participant characteristics, finger-tapping, VSRAD analysis value. Data described are as follows: patients’ ID; sex; age; education; MMSE-J, MoCA-J, Barthel Index, ADAS, RCPM, and FAB scores; measured values in finger-tapping; and VSRAD analysis value.
(XLSX)
