# Supplementary material for: Relationship between finger movement characteristics and brain voxel-based morphometry
Source: PLoS One. 2022 Oct 7;17(10):e0269351. doi: 10.1371/journal.pone.0269351 (PMC9543950; doi:10.1371/journal.pone.0269351)
Supplement: S5 Table — Displays the mean, standard deviation, and interquartile range for each indicator. (DOCX) [file pone.0269351.s005.docx]

**S5 Table.**

| Parameters for VSRAD | N=62 | | | |
| --- | --- | --- | --- | --- |
|  | mean | SD | correlation | p value |
| severity of medial temporal lobe atrophy |  |  |  |  |
| ・Total value | 1.87 | (0.76) | -.28 | 0.036 |
| ・Right side value | 1.86 | (0.90) | -.22 | 0.093 |
| ・Left side value | 1.69 | (1.02) | -.28 | 0.034 |
| ・Right side - Left side | 0.17 | (1.09) | -.05 | 0.698 |
| extent of gray matter atrophy | 5.55 | (2.75) | -.52 | <0.001 |
| extent of medial temporal lobe atrophy |  |  |  |  |
| ・Total value | 34.77 | (23.59) | -.27 | 0.042 |
| ・Right side value | 36.83 | (29.55) | -.25 | 0.063 |
| ・Left side value | 32.60 | (29.67) | -.18 | 0.176 |
| ・Right side - Left side | 4.23 | (35.80) | -.05 | 0.698 |
| Ratio of medial temporal lobe atrophy/gray matter atrophy | 7.34 | (6.00) | -.02 | 0.869 |
| Max in medial temporal lobe atrophy |  |  |  |  |
| ・Total value | 4.59 | (1.51) | -.27 | 0.043 |
| ・Right side value | 4.18 | (1.51) | -.24 | 0.070 |
| ・Left side value | 3.54 | (1.73) | -.32 | 0.014 |
| ・Right side - Left side | 0.65 | (1.70) | .12 | 0.357 |
| extent of white matter atrophy | 3.92 | (1.89) | -.30 | 0.020 |
